# Comprehensive analysis of PD‐L1 in non‐small cell lung cancer with emphasis on survival benefit, impact of driver mutation and histological types, and archival tissue

**DOI:** 10.1111/1759-7714.14216

**Published:** 2021-11-28

**Authors:** Chin‐Chou Wang, Kuo‐Tung Huang, Huang‐Chih Chang, Chia‐Cheng Tseng, Chien‐Hao Lai, Jui Lan, Ting‐Ting Liu, Chao‐Cheng Huang, Meng‐Chih Lin

**Affiliations:** ^1^ Divisions of Pulmonary & Critical Care Medicine, Department of Internal Medicine Kaohsiung Chang Gung Memorial Hospital, Chang Gung University College of Medicine Kaohsiung Taiwan; ^2^ Department of Respiratory Therapy, Kaohsiung Chang Gung Memorial Hospital Chang Gung University College of Medicine Kaohsiung Taiwan; ^3^ Department of Respiratory Care Chang Gung University of Science and Technology Chiayi Taiwan; ^4^ Department of Pathology, Chang Gung Memorial Hospital‐Kaohsiung Medical Center Chang Gung University College of Medicine Kaohsiung Taiwan; ^5^ Biobank and Tissue Bank and Department of Pathology Kaohsiung Chang Gung Memorial Hospital, Chang Gung University College of Medicine Kaohsiung Taiwan

**Keywords:** 22C3 IHC assay, ALK, EGFR, non‐small cell lung cancer (NSCLC), PD‐L1 expression

## Abstract

**Background:**

The aim of the study was to assess programmed death‐ligand‐1 (PD‐L1) expression in different histological types and gene mutation status of patients with non‐small cell lung cancer (NSCLC).

**Methods:**

A total of 4062 pathology‐confirmed lung cancer patients were retrospectively screened at Kaohsiung Chang Gung Memorial Hospital from November 2010 to June 2017. There were 699 NSCLC patients with confirmed PD‐L1 expression level retrospectively enrolled for analysis.

**Results:**

There was a trend of higher PD‐L1 expression in squamous cell carcinoma and adenosquamous cell carcinoma than in adenocarcinoma (*p* = 063). Significant higher PD‐L1 expression in *EGFR* wild‐type was noted (*p* < 0.001). No significant differences in PD‐L1 expression were found between ALK wild‐ and mutant types, but there seem was a trend of high PD‐L1 level noted in *ALK* mutation patients (*p* = 0.069). In *EGFR* mutation patients, a higher time to treatment failure (TTF) duration was observed in no PD‐L1 expression (*p* = 0.011). Longer tumor tissue storage time correlated with lower PD‐L1 expression in lung cancer (*p* < 0.001 for linear trend).

**Conclusions:**

There were a trend or significant differences in PD‐L1 expression between different histological types in NSCLC, different EGFR and ALK status, and different tumor tissue storage time. A higher survival benefit was observed in no PD‐L1 expression than with PD‐L1 expression in adenocarcinoma, *EGFR* and *ALK* mutation patients. We recommend that PD‐L1 assay should be performed as early as possible if tissue is available.

## Graphical abstract

A trend or significant differences in PD‐L1 expression between different histologic types in NSCLC, different EGFR status, and different ALK status, and different tumor tissue storage time. A higher survival benefit (TTF or OS) was observed in no PD‐L1 expression than with PD‐L1 expression in adenocarcinoma, *EGFR* mutation, and *ALK* mutation patients. PD‐L1 assay should be performed as early as possible if tissue is available.

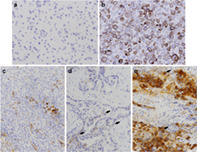

## INTRODUCTION

The immune system defends the body against infection and disease (including cancer). Certain inhibitory checkpoints drugs can help the immune system fight cancer. The immune system, by driven T lymphocytes and close regulation between inhibitory checkpoints and activating signals, plays an important role in controlling and eliminating cancer.^1^, ^2^, ^3^, ^4^, ^5^, ^6^, ^7^ Cytotoxic T lymphocyte antigen 4 (CTLA‐4) and programmed death‐1 (PD‐1) are the two main immune checkpoint receptors that when binding their ligand B7 and programmed death‐ligand‐1 (PD‐L1), respectively, determine the downregulation of the T cell effector functions, thus contributing to the maintenance of the tolerance to tumor cells.^3^, ^8^ The immune checkpoint inhibitors of PD‐1/PD‐L1 (anti–PD‐1/PD‐L1) are currently changing the approach to treatment of patients with advanced non‐small cell lung cancer (NSCLC). The FDA has released the approval of nivolumab (Opdivo, Bristol‐Myers Squibb), pembrolizumab (Keytruda, Merck Sharp and Dohme), and atezolizumab (Tecentriq, Genentech Oncology) for advanced NSCLC in patients previously treated with platinum‐based chemotherapy.^9^, ^10^, ^11^, ^12^ In addition, durvalumab (MEDI4736, AstraZeneca) and avelumab (MSB0010718C, Merck KGaA and Pfizer) are being investigated for the treatment of NSCLC.^13^, ^14^, ^15^

The expression of PD‐L1 has been reported in a number of human malignancies including NSCLC.^7^, ^16^ Immunoassays using different primary antibodies, assay formats, and scoring approaches have been reported to assess the prevalence of PD‐L1 positivity and the efficacy of treatment in NSCLC.^9^, ^10^, ^11^, ^12^, ^17^, ^18^ Reports in the literature have clearly shown that immune checkpoint inhibitors might represent an important therapeutic option for NSCLC patients. However, in spite of exciting overall treatment outcomes, a considerable number of patients failed to achieve long‐term clinical benefit.^9^, ^10^, ^11^, ^12^, ^14^, ^18^, ^19^ In the clinical trial of KEYNOTE‐001 and KEYNOTE‐010, Pembrolizumab had better efficacy in NSCLC patients with PD‐L1 expression level ≥ 50%.^11^, ^18^ Therefore, based on pembrolizumab series trials in NSCLC patients, PD‐L1 expression level might be a predictive biomarker for using pembrolizumab in NSCLC patients.^7^, ^16^

Although there have been previous reports regarding PD‐L1 expression in different histological and gene types of lung cancer, there are still few comprehensive studies on PD‐L1 expression of lung cancer in an endemic area with high epidermal growth factor receptor (*EGFR*) mutation such as Taiwan. In this study, we retrospectively reviewed the medical records of patients histologically or cytologically diagnosed with non‐small cell lung cancer from November 2010 to June 2017 at Kaohsiung Chang Gung Memorial Hospital (KCGMH) in Taiwan to assess PD‐L1 expression in different histological types and gene types of lung cancer.

## METHODS

The study was approved by the Institutional Review Board of Chang Gung Memorial Hospital, and the requirements for patient consent were waived (IRB: 201601146B0).

We retrospectively reviewed the medical records of patients histologically or cytologically diagnosed with lung cancer from November 2010 to June 2017 at Kaohsiung Chang Gung Memorial Hospital (KCGMH). KCGMH is a 2500 bed medical facility serving as a primary care and tertiary referral center in Kaohsiung, Taiwan. More than 600 new lung cancer patients each year have been documented and have received treatment in this hospital.

Data including basic demographic information, tumor histological type, epidermal growth factor receptor (EGFR) status, anaplastic lymphoma kinase (ALK) status, programmed death‐ligand 1 (PD‐L1) expression status, and formalin‐fixed paraffin‐embedded (FFPE) tumor tissue storage status were collected and analyzed. EGFR status was performed by EGFR RGQ PCR Kit (Qiagen). Automated immunohistochemical (IHC) study for ALK expression was performed in a Benchmark XT staining module (Ventana Medical Systems) on 5‐μm thick FFPE sections with D5F3 rabbit anti‐human CD246 monoclonal antibody. The anti–PD‐L1 antibody clone 22C3 (Merck) and a prototype IHC assay with a Dako Autostainer Link 48 platform (Agilent Technologies) was used to determine the PD‐L1 tumor proportion score (TPS). The PD‐L1 TPS was divided into no expression (<1%), low expression (1%–49%), and high expression (≥50%).^11^ Furthermore, tumor tissue storage time was also collected for analysis for archival tumor samples. The tumor tissue storage time was divided into four groups: <0.5 year, 0.5–2 years, 2–3.5 years, and ≥3.5 years. Staging was based on the American Joint Committee on Cancer (AJCC) seventh lung cancer TNM classification and staging system. Time to treatment failure (TTF) and overall survival (OS) were calculated to evaluate their efficacy. The TTF duration was defined as the interval from initiation of first‐line treatment to its discontinuation, and it could occur due to various reasons such as cancer progression, adverse events, patient choice, or patient death. Furthermore, the OS duration was calculated as the duration from osimertinib treatment initiation until patient death.

### Statistical analysis

Data (including age, sex, nodal stage, and EGFR mutation subtypes) were collected and analyzed using SPSS for Windows version 15.0 (SPSS Inc.). In descriptive statistics, data are presented as n (%) or median (interquartile range: Q1, Q3). Statistical significance of univariate analysis was determined by the Mann–Whitney U test and Kruskal‐Wallis test for continuous variables and chi‐square test for dichotomous variables. The log‐rank test was used to compare the survival distributions. Differences were considered significant when *p*‐value was <0.05.

## RESULTS

A total of 4062 pathology‐confirmed lung cancer patients were retrospectively screened at Kaohsiung Chang Gung Memorial Hospital from November 2010 to June 2017. There were 853 lung cancer patients assessed for PD‐L1 expression based on their specimens with anti–PD‐L1 antibody clone 22C3 IHC assay. A total of 731 cell lung cancer patients with PD‐L1 expression status available were retrospectively screened, and the remaining 122 were excluded due to inadequate tumor tissue (less than 100 tumor cells) for PD‐L1 analysis. Among the 731 lung cancer patients, there were 32 (4.4%) small cell lung cancer (SCLC) patients and 699 (95.6%) NSCLC patients. A total of 699 NSCLC patients were retrospectively enrolled for analysis in this study; there were 539 (77.1%) with adenocarcinoma, 66 (9.4%) with squamous cell carcinoma, 17 (2.4%) with adenosquamous carcinoma, and 77 (11.0%) with others (Table 2).

**FIGURE 1:**
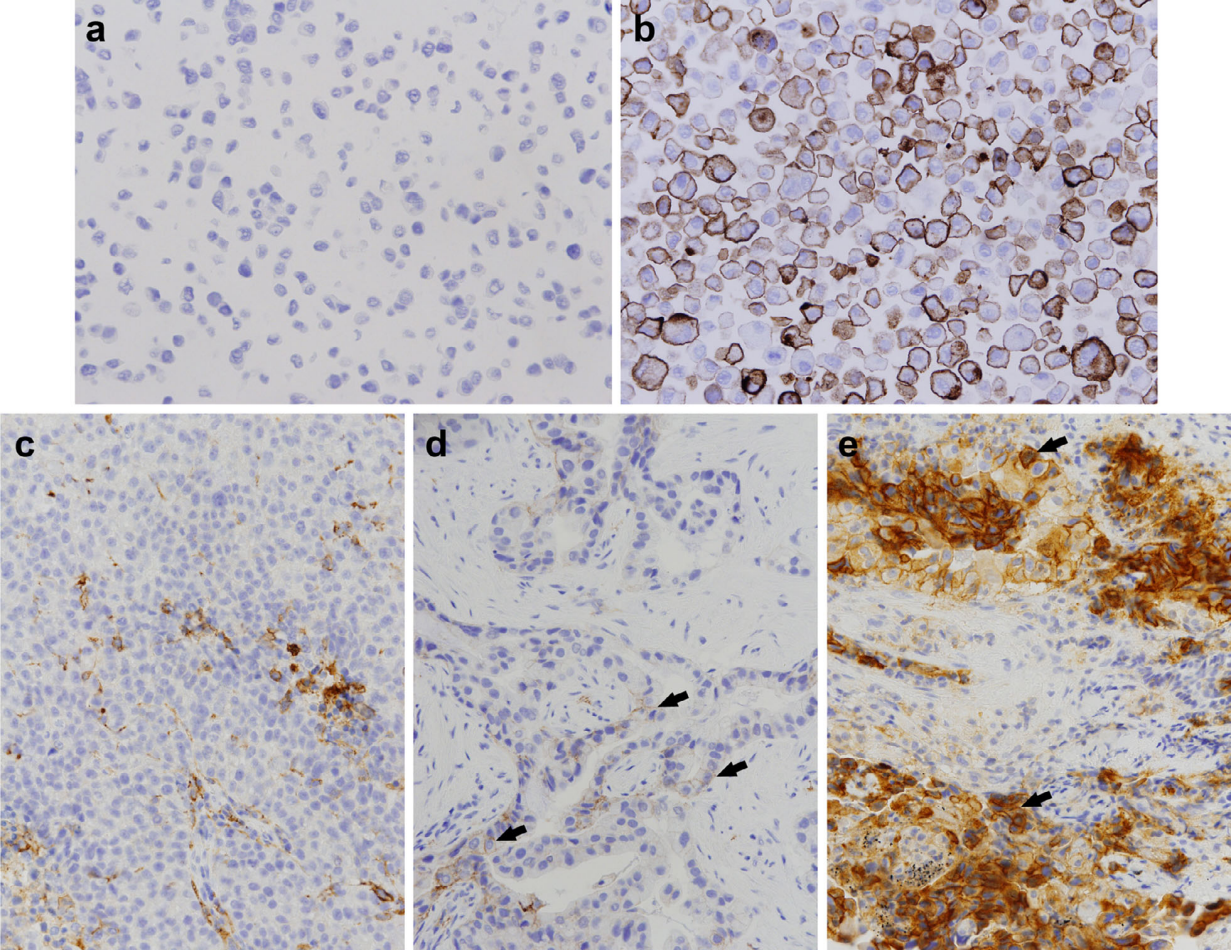
PD‐L1 immunohistochemical stain with 22C3 antibody. (a) The negative control cell line slide. (b) The positive control cell line slide with typically 80% positive cells. (c) A representative case of no expression for PD‐L1, tumor proportion score < 1%. (d) A representative case of low expression for PD‐L1, tumor proportion score 1 to 49%. (c) A representative case of high expression for PD‐L1, tumor proportion score ≥ 50%. Arrows indicate the positive tumor cells with membranous staining. Original magnification, 200x

Among the 699 NSCLC patients, there were 322 (46.1%) in the no expression group, 240 (34.3%) in the low expression group, and 137 (19.6%) in the high expression group (Table 1 and Figure 1). Furthermore, the demographic and clinical characteristics of 699 NSCLC patients are described in Table 1. The mean age of the patients was 64 (57, 72) years; 359 (51.4%) patients were men and 340 (48.6%) were women. Most of them were stage IV (526 [75.3%]) and non‐smoker (507[72.5%]).

**TABLE 1:** Demographic and clinical characteristics of all patients (*n* = 699)

Overall = 699	*n* (%)
Age (years)	
Median (Q1, Q3)	64 (57, 72)
Sex	
Male	359 (51.4%)
Female	340 (48.6%)
Stage	
I	45 (6.4%)
II	17 (2.4%)
IIIA	48 (6.9%)
IIIB	63 (9.0%)
IV	526 (75.3%)
Smoking status	
Non‐smoker	507 (72.5%)
Quit‐smoking	125 (17.9%)
Current‐smoking	67 (9.6%)
Types of specimens	
Bronchoscopy	352 (50.4%)
CT‐guided	63 (9.0%)
Thoracoscopy	158 (22.6%)
Pleural biopsy	40 (5.7%)
Others	86 (12.3%)
Mutation types	
EGFR (*n* = 500)	
Del19	133 (26.6%)
L858R	137 (27.4%)
Others	22 (4.4%)
Negative	208 (41.6%)
ALK (*n* = 450)	
Negative	427 (94.9%)
Positive	23 (5.1%)
PD‐L1 level	
No expression	322 (46.1%)
Low expression	240 (34.3%)
High expression	137 (19.6%)
1st‐line treatment (*n* = 586)	
TKI	318 (54.3%)
Chemotherapy	251 (42.8%)
Others	17 (2.9%)

*Note*: Data are presented as n (%), or median (Q1, Q3). Staging based on the AJCC seventh lung cancer TNM classification and staging system.

Abbreviation: ALK, anaplastic lymphoma kinase; EGFR, epidermal growth factor receptor; PD‐L1, programmed death‐ligand 1; TKI, tyrosine kinase inhibitors.

There was a trend of significant difference in PD‐L1 expression between tumor histologic types in NSCLC, showing higher PD‐L1 expression in squamous cell carcinoma and adenosquamous cell carcinoma than in adenocarcinoma (*p* = 063) (Table 2). Patients were predominantly female (*p* < 0.001), non‐smokers (*p* < 0.001), and received first‐line treatment with TKIs (*p* < 0.001) for adenocarcinoma than squamous cell carcinoma.

**TABLE 2:** PD‐L1 expression and clinical characteristics between different histological types

	Adenocarcinoma (*N* = 539, 77.1%)	Squamous cell carcinoma (*N* = 66, 9.4%)	Adenosquamous carcinoma (*N* = 17, 2.4%)	Others (*N* = 77, 11.0%)	Total (*N* = 699)	*p*‐value
Age (years)						0.098^a^
Median (Q1, Q3)	64 (57, 73)	64 (59, 74)	60 (54, 68)	63 (55, 70)	64 (57, 72)	
Sex						**<0.001** ^b^
Male	244 (45.3%)	54 (81.8%)	10 (58.8%)	51 (66.2%)	359 (51.4%)	
Female	295 (54.7%)	12 (18.2%)	7 (41.2%)	26 (33.8%)	340 (48.6%)	
Stage						**<0.001** ^b^
I	39 (7.2%)	3 (4.5%)	0 (0.0%)	3 (3.9%)	45 (6.4%)	
II	13 (2.4%)	0 (0.0%)	0 (0.0%)	4 (5.2%)	17 (2.4%)	
IIIA	24 (4.5%)	17 (25.8%)	2 (11.8%)	5 (6.5%)	48 (6.9%)	
IIIB	39 (7.2%)	10 (15.2%)	0 (0.0%)	14 (18.2%)	63 (9.0%)	
IV	424 (78.7%)	36 (54.5%)	15 (88.2%)	51 (66.2%)	526 (75.3%)	
Smoking status						**<0.001** ^b^
Non‐smoker	431 (80.0%)	17 (26.2%)	13 (76.5%)	46 (59.7%)	507 (72.5%)	
Quit‐smoking	69 (12.8%)	34 (51.5%)	3 (17.6%)	19 (24.7%)	125 (17.9%)	
Current‐smoking	39 (7.2%)	15 (22.7%)	1 (5.9%)	12 (15.6%)	67 (9.6%)	
PD‐L1 level						0.063^b^
No expression	263 (48.8%)	18 (27.2%)	5 (29.4%)	36 (46.7%)	322 (46.1%)	
Low expression	176 (32.7%)	31 (47.0%)	7 (41.2%)	26 (33.8%)	240 (34.3%)	
High expression	100 (18.6%)	17 (25.8%)	5 (29.4%)	15 (19.5%)	137 (19.6%)	
First‐line treatment						**<0.001** ^b^
TKI	302 (66.2%)	2 (4.0%)	6 (42.9%)	8 (12.1%)	318 (54.3%)	
Chemotherapy	144 (31.6%)	45 (90.0%)	8 (57.1%)	54 (81.8%)	251 (42.8%)	
Others	10.0 (2.2%)	3 (6.0%)	0 (0.0%)	4 (6.1%)	17.0 (2.9%)	

*Note*: Data are presented as n (%), or median (Q1, Q3). Staging based on the AJCC seventh lung cancer TNM classification and staging system. Post hoc test: In sex, *p* < 0.001 between adenocarcinoma and squamous cell carcinoma, *p* < 0.001 between adenocarcinoma and others. In stage, *p* = 0.005 between adenocarcinoma and squamous cell carcinoma, *p* = 0.005 between adenocarcinoma and others. In smoking status, *p* < 0.001 between adenocarcinoma and squamous cell carcinoma, *p* = 0.008 between adenocarcinoma and others, *p* = 0.004 between squamous cell carcinoma and others, *p* = 0.042 between adenosquamous carcinoma and others. In first line treatment, *p* < 0.001 between adenocarcinoma and squamous cell carcinoma, *p* < 0.001 between adenocarcinoma and others, *p* < 0.001 between squamous cell carcinoma and adenosquamous carcinoma, *p* < 0.001 between squamous cell carcinoma and others. Bold values indicate *p* < 0.05.

Abbreviations: EGFR, epidermal growth factor receptor; PD‐L1, programmed death‐ligand 1; TKI, tyrosine kinase inhibitors.

^a^ Kruskal‐Wallis test.

^b^ Chi‐square test.

For the correlation between *EGFR* mutation status and PD‐L1 expression in NSCLC, there were 292 (58.4%) out of 500 patients with *EGFR* mutation in our study cohort. Significantly higher PD‐L1 expression in *EGFR* wild‐type than in *EGFR* mutation was noted (*p* < 0.001) (Table 3), but no significant difference in PD‐L1 expression was found among different *EGFR* mutant forms (*p* = 0.207) (Table 4). In addition, there were 450 patients with available ALK status, and 23 (5.1%) patients had ALK rearrangement identified. No significant differences in PD‐L1 expression were found between *ALK* wild‐type and mutant type, but there was a trend of high PD‐L1 level noted in *ALK* mutation patients (*p* = 0.069) (Table 3).

**TABLE 3:** PD‐L1 expression and clinical characteristics with EGFR and ALK status

	EGFR				ALK			
	Negative (*N* = 208, 41.6%)	Positive (*N* = 292, 58.4%))	Total (*N* = 500)	*p*‐value	Negative (*N* = 427, 94.9%)	Positive (*N* = 23, 5.1%)	Total (*N* = 450)	*p*‐value
Age (years)				0.382^a^				0.542^a^
Median (Q1, Q3)	64 (57, 73)	65 (58, 73)	64 (57, 73)		65 (58, 73.25)	64 (57, 69.5)	65 (58, 73)	
Sex				**<0.001** ^b^				0.296^b^
Male	132 (63.5%)	109 (37.3%)	241 (48.2%)		232 (54.3%)	10 (43.5%)	242 (53.8%)	
Female	76 (36.5%)	183 (62.7%)	259 (51.8%)		195 (45.7%)	13 (56.5%)	208 (46.2%)	
Stage				**0.023** ^b^				0.522^b^
I	14 (6.7%)	11 (3.8%)	25 (5.0%)		35 (8.2%)	1 (4.3%)	36 (8.0%)	
II	5 (2.4%)	6 (2.1%)	11 (2.2%)		8 (1.9%)	2 (8.7%)	10 (2.2%)	
IIIA	14 (6.7%)	9 (3.1%)	23 (4.6%)		23 (5.4%)	0.0 (0.0%)	23 (5.1%)	
IIIB	25 (12.0%)	17 (5.8%)	42 (8.4%)		38 (8.9%)	0.0 (0.0%)	38 (8.4%)	
IV	150 (72.1%)	249 (85.2%)	399 (79.8%)		323 (75.6%)	20 (87.0%)	343 (76.2%)	
Smoking status				**<0.001** ^b^				0.300^b^
Non‐smoker	132 (63.5%)	249 (85.3%)	381 (76.2%)		305 (71.4%)	19 (82.6%)	324 (72.0%)	
Quit‐smoking	50 (24.0%)	29 (9.9%)	79 (15.8%)		79 (18.5%)	4 (17.4%)	83 (18.4%)	
Current‐smoking	26 (12.5%)	14 (4.8%)	40 (8%)		43 (10.1%)	0.0 (0.0%)	43 (9.6%)	
PD‐L1 level				**<0.001** ^b^				0.069^b^
No expression	68 (32.7%)	170 (58.2%)	238 (47.6%)		186 (43.6%)	5 (21.7%)	191 (42.4%)	
Low expression	78 (37.5%)	86 (29.5%)	164 (32.8%)		147 (34.4%)	9 (39.1%)	156 (34.7%)	
High expression	63 (29.8%)	36 (12.3%)	98 (19.6%)		94 (22.0%)	9 (39.1%)	103 (22.9%)	
First‐line treatment				**<0.001** ^b^				**0.007** ^b^
TKI	28 (13.5%)	276 (94.5%)	304 (60.8%)		241 (56.4%)	4 (17.4%)	245 (54.4%)	
Chemotherapy	172 (82.7%)	16 (5.5%)	188 (37.6%)		174 (40.7%)	19 (82.6%)	193 (42.9%)	
Others	8 (3.9%)	0 (0.0%)	8 (1.6%)		12 (2.8%)	0 (0.0%)	12 (2.7%)	

*Note*: Data are presented as n (%), or median (Q1, Q3). Staging based on the AJCC seventh lung cancer TNM classification and staging system. Bold values indicate *p* < 0.05.

Abbreviations: EGFR, epidermal growth factor receptor; PD‐L1, programmed death‐ligand 1; TKI, tyrosine kinase inhibitors.

^a^ Mann‐Whitney U test.

^b^ Chi‐square test.

**TABLE 4:** PD‐L1 expression and clinical characteristics with *EGFR* mutation status

	Del19 (*N* = 133)	L858R (*N* = 137)	Others (*N* = 22)	Total (*N* = 292)	*p*‐value
Age (years)					0.078^a^
Median (Q1, Q3)	64 (54, 71)	66 (60, 74)	67 (65, 71)	65 (58, 73)	
Sex					0.193^b^
Male	52 (39.1%)	53 (38.7%)	4 (18.2%)	109 (37.3%)	
Female	81 (60.9%)	84 (61.3%)	18 (81.8%)	183 (62.7%)	
Stage					0.073^b^
I	5 (3.8%)	5 (3.6%)	1 (4.5%)	11 (3.8%)	
II	1 (0.8%)	5 (3.6%)	0 (0.0%)	6 (2.1%)	
IIIA	1 (0.8%)	6 (4.4%)	1 (4.5%)	8 (2.7%)	
IIIB	9 (6.8%)	6 (4.4%)	1 (4.5%)	16 (5.5%)	
IV	117 (88.0%)	115 (83.9%)	19 (86.4%)	251 (86.0%)	
Smoking status					0.517^b^
Non‐smoker	110 (82.7%)	121 (88.3%)	18 (81.8%)	249 (85.3%)	
Quit‐smoking	16 (12.0%)	12 (8.8%)	1 (4.5%)	29 (9.9%)	
Current‐smoking	7 (5.3%)	4 (2.9%)	3 (13.6%)	14 (4.8%)	
PD‐L1 level					0.207^b^
No expression	77 (57.9%)	79 (57.7%)	13 (59.1%)	169 (57.9%)	
Low expression	33 (24.8%)	46 (33.6%)	8 (36.4%)	87 (29.8%)	
High expression	23 (17.3%)	12 (8.8%)	1 (4.5%)	36 (12.3%)	
First‐line treatment					0.296^b^
TKI	127 (95.5%)	127 (92.7%)	22 (100.0%)	276 (94.5%)	
Chemotherapy	6 (4.5%)	10 (7.3%)	0 (0.0%)	16 (5.5%)	

*Note*: Data are presented as n (%), or median (Q1, Q3). Staging based on the AJCC seventh lung cancer TNM classification and staging system.

Abbreviations: EGFR, epidermal growth factor receptor; PD‐L1, programmed death‐ligand 1; TKI, tyrosine kinase inhibitors.

^a^ Kruskal‐Wallis test.

^b^ Chi‐square test.

The correlation of PD‐L1 expression and survival benefit of 699 NSCLC patients are described in Table 5. There were significant differences in TTF (*p* < 0.001) and OS (*p* = 0.029) observed between different PD‐L1 expression levels in all patients. In *EGFR* mutation patients, a higher TTF duration was observed in no PD‐L1 expression than in low PD‐L1 expression (*p* = 0.007) and high PD‐L1 expression (*p* = 0.011); on the contrary, no significant difference in TTF was observed between different PD‐L1 expression level in *ALK* mutation patients (*p* = 0.266). Furthermore, no significant difference in OS was observed between different PD‐L1 expression level in both *EGFR* and *ALK* mutation patients.

**TABLE 5:** The correlation of PD‐L1 expression and survival benefit

	No expression	Low expression	High expression	
**All patients**	***N* = 205 (43.16%)**	***N* = 166 (34.95%)**	***N* = 104 (21.89%)**	
TTF (months)	12.16 (5.33, 24.23)	8.48 (3.42, 15.365)	5.225 (2.228, 11.57)	***p*** ^a^ **< 0.001**
OS (months)	74.63 (63.75, NA)	49.38 (30.97, NA)	47.21 (28.31, NA)	***p*** ^b^ **= 0.029**
**Adenocarcinoma**	***N* = 197 (51.98%)**	***N* = 114 (30.08%)**	***N* = 68 (17.94%)**	
TTF (months)	14.5 (6.05, 25.775)	10.72 (4.55, 18.05)	5.9 (2, 12.718)	***p*** ^a^ **< 0.001**
OS (months)	75.45 (66.87, NA)	49.38 (30.97, NA)	47.21 (28.31, NA)	***p*** ^b^ **= 0.009**
**Squamous cell carcinoma**	***N* = 8 (26.67%)**	***N* = 17 (56.67%)**	***N* = 5 (16.67%)**	
TTF (months)	5.49 (3.368, 5.693)	4.075(1.968, 6.395)	4.87 (3.65, 8.12)	*p* ^a^ = 0.422
OS (months)	50.17 (34.4, NA)	31.04 (18.97, NA)	NA (21.76, NA)	*p* ^b^ = 0.784
**EGFR negative**	***N* = 36 (30.77%)**	***N* = 48 (41.03%)**	***N* = 33 (28.2%)**	
TTF (months)	5.935 (3.218, 10.485)	5.34 (2.375, 12.1425)	3.78 (0.46, 7.2)	*p* ^a^ = 0.085
OS (months)	NA (NA, NA)	40.96 (25.15, NA)	14.93 (7.46, NA)	P^b^ = 0.065
**EGFR positive**	***N* = 124 (58.49%)**	***N* = 63 (29.72%)**	***N* = 25 (11.79%)**	
TTF (months)	18.97 (9.668, 32.4)	12.03 (6.44, 19.63)	8.94 (2.76, 18.12)	***p*** ^a^ **< 0.001**
OS (months)	74.63 (66.87, NA)	NA (30.97, NA)	NA (28.31, NA)	*p* ^b^ = 0.665
**ALK negative**	***N* = 106 (40.61%)**	***N* = 96 (36.78%)**	***N* = 59 (22.6%)**	
TTF (months)	11.59 (5.423, 24.678)	9.5 (3.63, 16.725)	5 (2.385, 9.24)	***p*** ^a^ **< 0.001**
OS (months)	67.2 (52.44, NA)	51.09 (25.61, NA)	48.79 (21.76, NA)	*p* ^b^ = 0.298
**ALK positive**	***N* = 4 (30.77%)**	***N* = 4 (30.78%)**	***N* = 5 (38.46%)**	
TTF (months)	5.935 (4.313, 7.64)	2.335 (1.038, 3.77)	9.21 (4.87, 12.56)	*p* ^a^ = 0.266
OS (months)	34.42 (34.42, NA)	17.72 (NA, NA)	8.61 (0.33, NA)	*p* ^b^ = 0.78
**EGFR Del19**	***N* = 60 (58.82%)**	***N* = 26 (25.49%)**	***N* = 16 (15.69%)**	
TTF (months)	19.46 (9.99, 28.9)	11.115 (6.778, 16.59)	13.66 (3.243, 18.53)	*p* ^a^ = 0.062
OS (months)	75.45 (53.49, NA)	NA (32.02, NA)	NA (28.31, NA)	*p* ^b^ = 0.9
**EGFR L858R**	***N* = 52 (55.91%)**	***N* = 33 (35.48%)**	***N* = 8 (8.6%)**	
TTF (months)	16.85 (9.24, 27.813)	12.39 (8.02, 21.57)	5.92 (2.638, 11.273)	*p* ^a^ = 0.06
OS (months)	67.2 (48.23, NA)	53.62 (26.33, NA)	NA (15.19, NA)	*p* ^b^ = 0.795

*Note*: Data are presented as n (%), or median (Q1, Q3). Staging based on the AJCC seventh lung cancer TNM classification and staging system. Post hoc test: In all patients, *p* = 0.002 in TTF between no expression andlLow expression, *p* < 0.001 in TTF between no expression and high expression, *p* = 0.023 in TTF between low expression and high expression, *p* = 0.017 in OS between no expression and low expression, *p* = 0.024 in OS between no expression and high expression, *p* = 0.857 in OS between low expression and high expression. In adenocarcinoma, *p* = 0.014 in TTF between no expression and Low expression, *p* < 0.001 in TTF between no expression and high expression, *p* = 0.012 in TTF between low expression and high expression, *p* = 0.004 in OS between no expression and low expression, *p* = 0.017 in OS between no expression and high expression, *p* = 0.979 in OS between low expression and high expression. In EGFR negative, *p* = 0.039 in OS between no expression and low expression, *p* = 0.023 in OS between no expression and high expression, *p* = 0.585 in OS between low expression and high expression. In EGFR positive, *p* = 0.007 in TTF between no expression and low expression, *p* = 0.011 in TTF between no expression and high expression, *p* = 0.46 in TTF between low expression and high expression. In ALK negative, *p* = 0.141 in TTF between no expression and low expression, *p* < 0.001 in TTF between no expression and high expression, *p* = 0.008 in TTF between low expression and high expression. Bold values indicate *p* < 0.05.

Abbreviations: EGFR, epidermal growth factor receptor; OS, overall survival; PD‐L1, programmed death‐ligand 1; TKI, tyrosine kinase inhibitors; TTF, yime to treatment failure.

^a^ Kruskal‐Wallis test.

^b^ Log‐rank test.

Finally, we also assessed the impact of tumor tissue storage time to PD‐L1 expression in lung cancer. There was significant change in PD‐L1 expression between different tumor tissue storage time in lung cancer (*p* < 0.001 for linear trend), showing a lower PD‐L1 expression with prolonged tumor tissue storage (Table 6).

**TABLE 6:** The correlation of PD‐L1 expression with tumor tissue storage time in lung cancer

Total, *n* = 699	No expression	Low expression	High expression	*p*‐value
<0.5 year (*n* = 443)	167 (37.7%)	164 (37.0%)	112 (25.3%)	**<0.001** ^a^
0.5–2 years (*n* = 159)	90 (56.6%)	40 (25.2%)	29 (18.2%)	
2–3.5 years (*n* = 48)	28 (58.3%)	16 (33.3%)	4 (8.3%)	
≥3.5 years (*n* = 49)	34 (69.4%)	11 (22.4%)	4 (8.2%)	

*Note*: Tumor tissue storage time was divided into four groups: <0.5 year, 0.5–2 years, 2–3.5 years, and ≥3.5 years. Others means tissue from other than lung including liver, brain, bone, or lymph node. Post hoc test: high expression versus no expression (*p* < 0.001), low expression versus no expression (*p* < 0.001), low expression versus high expression (*p* = 0.295). Bold value indicate *p* < 0.05.

Abbreviations: PD‐L1, programmed death‐ligand 1.

^a^ Chi‐square test, *p* < 0.001 for linear trend.

## DISCUSSION

In this study, we evaluated PD‐L1 expression on 655 enrolled lung cancer patients with anti–PD‐L1 antibody clone 22C3 IHC assay. Two previous clinical trials, KEYNOTE‐001 and KEYNOTE‐010, also analyzed PD‐L1 expression with the same platform. The KEYNOTE‐001 trial had enrolled 824 patients and the KEYNOTE‐010 enrolled 2222 patients for PD‐L1 testing.^11^, ^18^

There was a significant difference in PD‐L1 expression between our study and KEYNOTE‐010 (*p* < 0.001) and between KEYNOTE‐001 and KEYNOTE‐010 (*p* = 0.005) (Table 7). These results may have been caused by a difference in terms of tissue storage between prospective clinical trials and our retrospective study, distinct PD‐L1 expression in different populations or genetic background, and selection bias in screen procedures for clinical trials.

**TABLE 7:** The comparison of PD‐L1 expression between our study and KEYNOTE‐001 and ‐010

Total, *n* = 655	Total screen	Enrolled	No expression	Low expression	High expression	*p*‐value
Our study	4062	699	322 (46.1%)	220 (34.3%)	137 (19.6%)	**<0.001**
KEYNOTE‐001	1143	824	323 (39.2%)	310 (37.6%)	191 (23.2%)	
KEYNOTE‐010	2699	2222	747 (33.6%)	842 (37.9%)	633 (28.5%)	

*Note*: Our study versus KEYNOTE‐001: *p* = 0.118. Our study versus KEYNOTE‐010: *p* < 0.001. KEYNOTE‐001 versus KEYNOTE‐010: *p* = 0.005. Bold value indicate *p* < 0.05.

Abbreviations: PD‐L1, programmed death‐ligand 1.

There was significant difference in PD‐L1 expression detected between different tumor tissue storage time for lung cancer in our study (*p* < 0.001 for linear trend, Table 6). The new tissue sample appeared to have higher PD‐L1 expression than archival tissue sample (Table 6). As our hospital is located in a subtropical area in a humid, warm to hot climate, we routinely keep our archival FFPE tissue blocks in an air‐conditioned room of 24–26°C. However, long‐term storage might decrease immunoreactivity of tissue for IHC study. A small series of PD‐L1 study in 58 NSCLC has shown fading with time of PD‐L1 immunoreactivity, which is in agreement with our results.^20^ Another issue of concern is tissue availability for current personalized medicine. For most lung cancer patients, the tumor tissue samples for diagnosis are usually small with a limited number of tumor cells obtained from either fibrobronchoscopy or chest CT‐guided biopsy. For such a small biopsy sample, tissue conservation strategies are crucial for further molecular analysis and PD‐L1 evaluation.^21^ Accordingly, to prevent tissue exhaustion and increase detection sensitivity, we recommend that PD‐L1 assay should be performed as early as possible if the tissue is available. In addition, a comparison of the prevalence of PD‐L1 protein positivity in a renal cancer cohort with fresh frozen tissue versus in FFPE tissue demonstrated a higher PD‐L1 positivity rate in the cohort with fresh frozen tissue (37% vs. 24%, respectively).^22^, ^23^, ^24^ The decreased positivity rate in the FFPE tissue may be caused by PD‐L1 protein denaturation with formalin fixation and a loss in PD‐L1 antigenicity.^25^ The correlation of types of specimens with tumor tissue storage time is described in Table 8. There was high proportion tissue obtained by chest CT‐guided biopsy or video‐assisted thoracoscopic surgery (VATS).

**TABLE 8:** The correlation of types of specimens with tumor tissue storage time

	Bronchoscopy	CT‐guided	VATS	Pleural biopsy	Others	*p*‐value
<0.5 year (*n* = 443)	226 (64.2%)	49 (77.8%)	127 (80.9%)	15 (36.6%)	26 (30.2%)	**<0.001** ^a^
0.5‐2 years (*n* = 159)	95 (27.0%)	11 (17.5%)	21 (13.4%)	10 (24.4%)	22 (25.6%)	
2–3.5 years (*n* = 48)	15 (4.3%)	2 (3.2%)	5 (3.2%)	7 (17.1%)	19 (22.1%)	
≥3.5 years (*n* = 49)	16 (4.5%)	1 (1.6%)	4 (2.5%)	9 (22.0%)	19 (22.1%)	
Total	352 (100.0%)	63 (100.0%)	157 (100.0%)	41 (100.0%)	86 (100.0%)	

*Note*: Tumor tissue storage time was divided into four groups: <0.5 years, 0.5–2 years, 2–3.5 years, and ≥3.5 years. Others means tissue form other than lung; including liver, brain, bone, or lymph node. Bold value indicate *p* < 0.05.

Abbreviations: CT, computed tomography; VATS, video‐assisted thoracoscopic surgery.

^a^ Chi‐square test.

Our finding that 18.6% of adenocarcinoma (Table 2) had a high PD‐L1 expression (TPS ≥ 50%) is lower than previous reports which used the same antibody and platform in larger cohorts. A PD‐L1 TPS of at least 50% was reported in 24.9% to 30.2% of advanced NSCLCs in the phase I to III trials (KEYNOTE‐001, KEYNOTE‐010, and KEYNOTE‐024) of pembrolizumab.^11^, ^18^, ^26^ Our data is also lower than another study of Beth Israel Deaconess Medical Center, Harvard Medical School) which showed 29.6% of adenocarcinoma had a PD‐L1 TPS of at least 50%.^27^ These differences might be due to the different *EGFR* mutation distribution between East Asia and West areas.^28^ There was a higher *EGFR* mutation rate in East Asia area than in West area, as shown in our study cohort that the EGFR mutation rate was 58.4%, and there was higher PD‐L1 expression in *EGFR* wild‐type than *EGFR* mutation type.^26^, ^27^ It was partly demonstrated by the increased percentage of high PD‐L1 expression (TPS ≥ 50%) up to 29.8% of NSCLC with wild‐type EGFR in our study (Table 3). In addition, our study cohort was a retrospective study with longer tissue storage in a part of archival tissue samples, and a prolonged tissue storage could lower PD‐L1 expression as previously mentioned.

In one of the largest published screening cohorts for PD‐L1 using anti–PD‐L1 antibody clone 22C3 IHC assay to date (i.e., that in the KEYNOTE‐024 trial), the frequency of overlap between common driver oncogene aberrations (i.e., in EGFR or ALK) and a PD‐L1 expression level of at least 50% was just 6% (30 of 500).^26^ There was higher PD‐L1 expression level in *EGFR* wild‐type than *EGFR* mutation type in our study (*p* < 0.001, Table 3). However, there was no significant difference in PD‐L1 between *ALK* wild‐type and *ALK* mutation type (*p* = 0.069, Table 3). The small sample size might not reflect the real condition of PD‐L1 expression level in ALK mutation patients in our study.

PD‐L1 expression in lung cancer could be heterogeneous and dynamic. Therefore, the consistency, reliability and feasibility to test PD‐L1 expression on a single biopsy specimen as a reference for immuno‐oncology treatment remains controversial.

It has been reported that *EGFR* mutation status is related to PD‐L1 expression, with lower PD‐L1 expression level noted in adenocarcinoma patients with *EGFR* mutation.^29^, ^30^ In another study, the author found that EGFR‐TKIs directly inhibit tumor cell vitality, and also indirectly strengthen antitumor immunity by downregulating PD‐L1.^31^ This could explain why there was better TTF duration in adenocarcinoma patients with *EGFR* mutation in our study.

Furthermore, our retrospective study has several limitations. First, this study was conducted at a single medical center, and the patient population may be biased by patient selection and referred pattern. Second, this study was a retrospective survey, which not only resulted in incomplete data for some patients, but also did not control for the clinical courses of all lung cancer patients. Hence, further prospective investigations should be conducted to further validate the findings. Despite these limitations, this study provides relatively valuable data regarding the different survival benefit between subgroup and the significant decrease in PD‐L1 expression along with increase in tumor tissue storage time.

In conclusion, we have shown there was a trend or significant differences in PD‐L1 expression between different histological types in NSCLC, different EGFR status, and different ALK status, and different tumor tissue storage time; a higher survival benefit (TTF or OS) was observed in no PD‐L1 expression than in with PD‐L1 expression in adenocarcinoma, *EGFR* mutation, and *ALK* mutation patients. Furthermore, we recommend that PD‐L1 assay should be performed as early as possible if the tissue is available.

## CONFLICT OF INTEREST

The authors state that that there are no potential conflicts of interest.
